# Verification of impact of morning showering and mist sauna bathing on human physiological functions and work efficiency during the day

**DOI:** 10.1007/s00484-014-0932-3

**Published:** 2014-11-12

**Authors:** Soomin Lee, Hiroko Fujimura, Yoshihiro Shimomura, Tetsuo Katsuura

**Affiliations:** 1 Center for Environment, Health and Field Sciences, Chiba University, 6-2-1 Kashiwanoha, Kashiwa, Chiba, 277-0882 Japan; 2 Urban Research Institute, Tokyo Gas Co., Tokyo, Japan; 3 Graduate School of Engineering, Chiba University, Chiba12, Japan

**Keywords:** Mist sauna, Physiological function, Work efficiency, Morning bathing

## Abstract

Recently, a growing number in Japan are switching to taking baths in the morning (morning bathing). However, the effects of the morning bathing on human physiological functions and work efficiency have not yet been revealed. Then, we hypothesized that the effect of morning bathing on physiological functions would be different from those of night bathing. In this study, we measured the physiological functions and work efficiency during the day following the morning bathing (7:10–7:20) including showering, mist sauna bathing, and no bathing as a control. Ten male healthy young adults participated in this study as the subjects. We evaluated the rectal temperature (Tre), skin temperature (Tsk), heart rate (HR), heart rate variability (HRV), blood pressure (BP), the relative power density of the alpha wave (α-wave ratio) of electroencephalogram, alpha attenuation coefficient (AAC), and the error rate of the task performance. As a result, we found that the HR after the mist sauna bathing was significantly lower than those after no bathing rest 3 (11:00). Furthermore, we verified that the α-wave ratio of the Pz after the mist sauna bathing was significantly lower than those after no bathing during the task 6 (15:00). On the other hand, the α-wave ratio of the Pz after the mist sauna bathing was significantly higher than those after showering during the rest 3 (11:00). Tsk after the mist sauna bathing was higher than those after the showering at 9:00 and 15:00. In addition, the error rate of the task performance after the mist sauna bathing was lower than those after no bathing and showering at 14:00. This study concludes that a morning mist sauna is safe and maintains both skin temperature compared to other bathing methods. Moreover, it is presumed that the morning mist sauna bathing improves work efficiency comparing other bathing methods during the task period of the day following the morning bathing.

## Introduction

It is a Japanese custom to bathe daily for cleanliness, warmth, and relaxation (Tochihara 1999). Bathing that involves washing grime from the body and face with soap while immersed in a hot bath can cause major changes to the physiological function of the skin (Berardesca et al. 1995; Okuda et al. 2002) and to thermoregulation (Hashiguchi et al. 2002).

Many previous studies have reported the effects of bathing circumstances, method, style, postures, etc., on human physiological and psychological functions. Miwa et al. (1994, 1998, 1999), concluded that bathing can induce remarkable changes in the cardiovascular system by increasing core temperature through immersion in hot water (40 °C) for more than 10 min. With regard to posture while bathing, Onaka et al. (1995) reported that blood pressure (BP) decreased rapidly during reclined or “sink” bathing in contrast to sitting or standing showers, in which BP increased. Moreover, mean skin temperature (Tsk) during sink bathing was significantly higher than during standing or seated showers. In terms of style of bathing, Johnston et al. (1981) found higher oxygen consumption and electrocardiographic changes during showering than during a full immersion bath. Furthermore, Mizuno et al. (2010) found that heart rate was higher, muscle stiffness at the waist was lower, and plasma cortisol levels tended to be lower after mild-stream bathing than after a full immersion bath. Previously, we compared the effects of the following bathing methods: full immersion bath; shower; mist sauna; and no bathing. We verified that full immersion baths and mist saunas are effective in facilitating recovery from muscular fatigue (Lee et al. 2012).

Traditionally, Japanese people bathe at night. However, according to 2011 internal study of Urban Research Institute (Tokyo Gas Co., Ltd. Japan), it was reported that many people in Japan are switching to taking a shower in the morning (more than 4 times/week: 15.8 %, 1–3 times/week: 23.2 % in summer, *n* = 3,267). Therefore, we hypothesized that the effect of morning showering on physiological functions would be different from those of night bathing. In the interest of public health, it is important to establish the physiological differences and work efficiency in terms of morning showering. In addition, the mist sauna bathing is prevalent a part of modern life in Japan. Unfortunately, physiological data on the mist sauna bathing effect were limited. Hence, in the present study, we investigated the effects of morning showering and mist sauna bathing on human physiological functions and work efficiency during the day; bathing conditions included showering, mist sauna, and no bathing.

## Methods

### Subjects

Ten healthy male students (24 ± 3 years, 174 ± 6.1 cm, 70 ± 7.6 kg) participated in the study. Subjects were instructed to refrain from intense physical activity on the day prior to the experiment and from drinking caffeinated beverages and smoking cigarettes for 2 h immediately preceding the experiment. Subjects were asked to maintain their regular sleep-wake cycle. Written informed consent was obtained from every subject. This experiment was approved by the bioethics committee of the Graduate School of Engineering, Chiba University.

### Experimental conditions

Three different bathing conditions were investigated: showering (S), mist sauna (M), and no bathing (C) as the control. For each condition, subjects spent a total of 3 days and two nights in the laboratory. The three conditions were as follows: in the mist-sauna condition (TOKYO GAS Co., Ltd., ABD-4107BCSK-M), subjects were splashed with a mist of fine drops of warm (40 °C) water; in the showering condition, subjects were drenched with warm (40 °C) water while in a seated position outside the bathtub; in the no bathing condition, subjects sat on a chair outside the bathtub in a room heated to (26 °C), similar to the showering condition. The three experiments were conducted at the same time of day on three separate days. The protocol consisted of three experiments on three different days at 1-week intervals. The order of the three conditions was counterbalanced between subjects. Subjects were directed to wear only shorts and t-shirts in the pre-room and only shorts in the bathroom.

### Experimental environment

The air temperature and relative humidity of the laboratory were controlled at 26 °C and 50 %, respectively. The laboratory consisted of a pre-room and bathroom. Subjects were in the pre-room at all times except for urination, bowel movements, and bathing. Lighting of the pre-room was controlled as follows: 7:00 to 9:00 at 300 lx, 9:00 to 17:00 at 800 lx, and 17:00 to 24:00 at 100 lx. Subjects bathed at 7:00 on the second day.

### Procedure

**Fig. 1:**
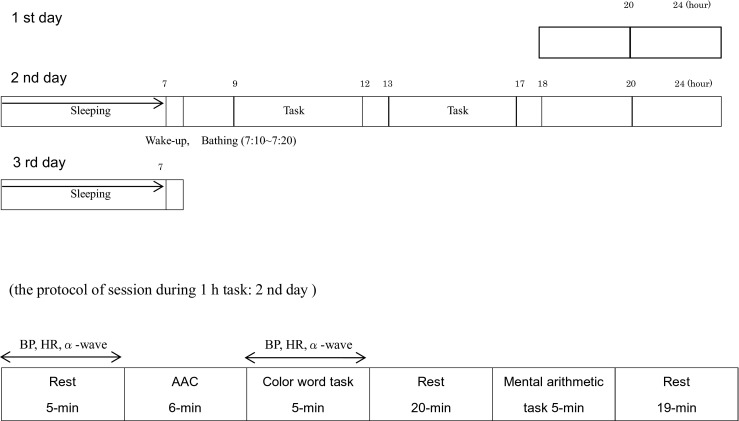
The protocol of the experiment

The first night, the subjects entered the pre-room at 19:00; from that point forward, their skin temperature (Tsk) and rectal temperature (Tre) were monitored with thermistors until the end of the experiment. They slept from 24:00. On the second day, they got up at 7:00 and bathed for 10 min beginning at 7:10. We defined subjects’ protocol during 1-h tasks as a session. Each session between 9:00 and 17:00 proceeded as follows: subjects were asked to relax for 5 min; we then conducted the alpha attenuation coefficient (AAC) for a 6 min and the Stroop’s color-word task for 5 min; subjects then rested for 20 min; following rest, subjects conducted a mental arithmetic task for 5 min, then rested again for 19 min. On the third day, subjects got up at 7:00 and were finished with measurements at 7:30. The experimental procedure was shown Fig. 1.

### Mental tasks

In the previous studies, the Stroop’s color-word conflict task (CWT) was widely used as a mental task (Swan et al. 2014; Ergen et al. 2014; Yokoi et al. 2003). In this study, the CWT and mental arithmetic task were used as mental tasks. The CWT was a computerized color-word interference task. The Japanese words “green,” “yellow,” “black,” “red,” “blue,” and “purple” were randomly flashed in the center of a computer display. While each color word was also written in one of these six colors, the name of the word and color of the word were different. The task was to use a mouse to click a button that corresponded to the name of the color within 3 s. If subjects exceeded the 3-s limit, an alarm would sound and a new word would be automatically presented (Liu et al. 2007). The mental arithmetic task consisted of two-digit number subtraction. However, we did not check the results of this task. It was simply used to prevent subjects from sleeping.

### Measurements

Electroencephalogram (EEG) was recorded with Ag/AgCl electrodes affixed with electrode paste at the Fz, Cz, and Pz electrode sites of the international 10–20 system. Linked earlobes were used as a reference with a forehead ground. A bipolar electrooculogram (EOG) was recorded with electrodes placed above and below the left eye. The EEG and EOG were amplified by appropriate devices (EEG100B and EOG100B, BIOPAC Systems). EEGs were fast Fourier transformed for each 5.12 s of data, not including artifacts such as ocular movement. We then obtained the relative power density of the alpha wave (8–13 Hz/4–30 Hz). The AAC (alpha band power when eyes are closed/alpha band power when eyes are open) was calculated and analyzed (Yoto et al. 2007).

Electrocardiogram (ECG) was recorded using an amplifier (ECG 100B, BIOPAC Systems) and digitized to derive heart rate variability (HRV) and heart rate (HR) using signal processing software (Mathcad, PTC). The high frequency (HF) component and low frequency (LF) component were integrated at 0.05–0.15 and 0.15–0.40 Hz of the power spectra, respectively (Pomeranz et al. 1985; Pagani et al. 1986). Sympathetic nervous activity (LF / HF) and parasympathetic nervous activity (HF / (LF + HF)) were calculated. All signals of the physiological indexes were converted from analog to digital at a 1-kHz sampling rate (MP150, BIOPAC Systems) and stored in a computer. In addition, Tre and Tsk were measured at four sites (thigh, foot, forearm, and chest) with thermistors. The thermistor probe for Tre was inserted 10 cm beyond the anal sphincter. These temperature data were recorded every 2 s by a data logger (LT-8; Gram Corporation). The blood pressure was measured intermittently in the upper arm (UM-15P; Parama-Tech).

### Statistical analyses

The values of each physiological parameter from the second day were used. Changes in the averages for BP, HR, the relative power density of the alpha during the 5-min rest, and the average of the 6-min AAC were calculated by subtracting from the values obtained during the 9:00∼10:00 session. Furthermore, the average of the color-word task was subtracted from the average of the 9:00∼10:00 session. On the other hand, changes of the Tsk and Tre were calculated by subtracting measures from the value of the 6:50∼7:00 (the average of the 5 min). For the physiological parameters, we conducted a one-way repeated-measure ANOVA (bathing method factor). When a significant *F* value was found, we used the Holm method as a post hoc test. All statistical analyses were performed using SPSS 18.0J (SPSS, Japan). A probability level of 0.05 was considered statistically significance. Data are shown as mean ± SD unless otherwise stated.

## Results

**Fig. 2:**
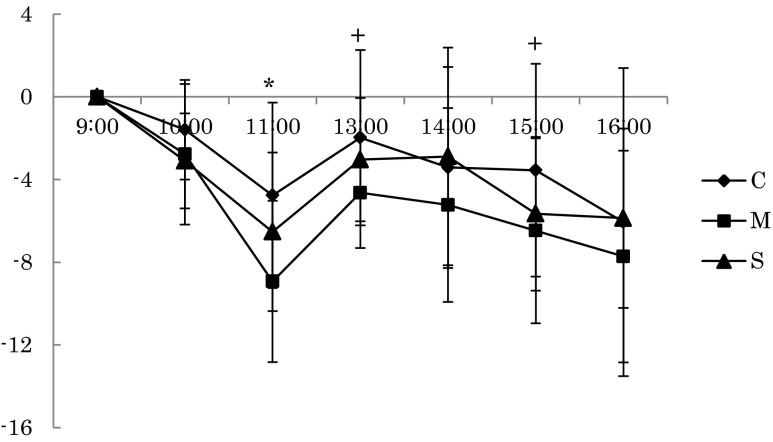
The changes of HR in the rest period (*p < 0.05, +*p* < 0.1)

**Fig. 3:**
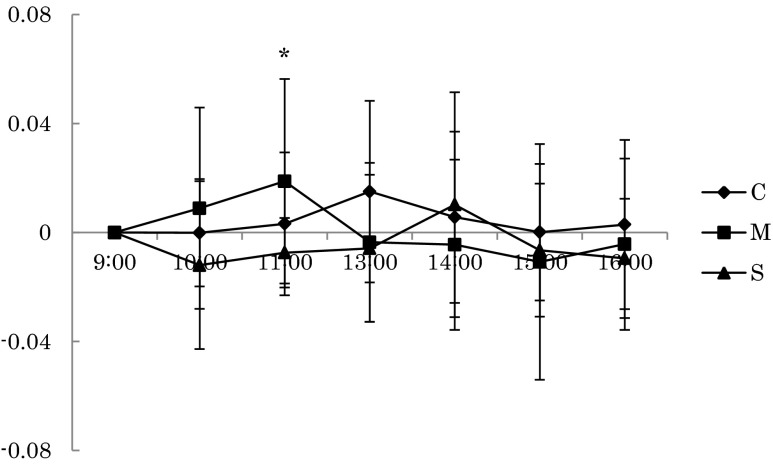
The changes of the relative power density of the alpha wave at Pz during rest (*p < 0.05)

**Fig. 4:**
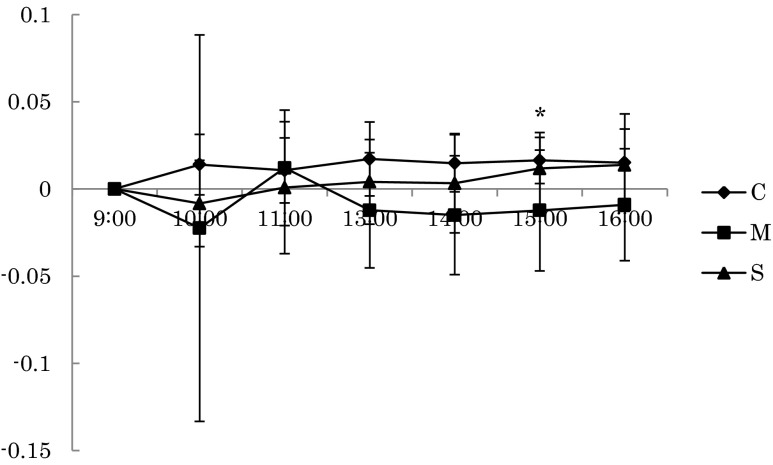
The changes of the relative power density of the alpha wave at Pz during task (*p < 0.05)

**Fig. 5:**
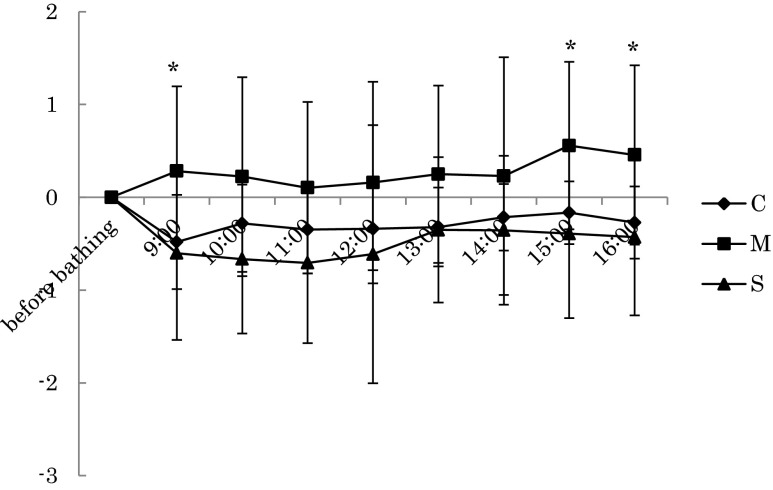
The changes of the Tsk of left chest (*p < 0.05)

**Fig. 6:**
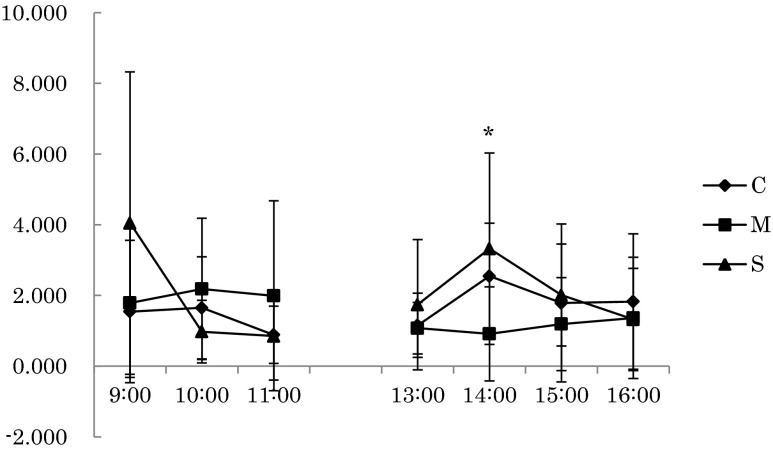
The result of error rate in the color-word task (*p < 0.05)

The results of each of the three conditions from 9:00 to 17:00 on the second day were as follows. HR during rest in the mist sauna condition was significantly lower than in the no bathing condition at the 11:00 [*F(*2,18) = 5.428, *p* = 0.014]. Furthermore, HR during rest in the mist sauna condition tended to be lower than that of the no bathing condition at 13:00 and 15:00 (Fig. 2). Meanwhile, the relative power density of the alpha wave of Cz during the task in the showering condition was significantly lower than that of no the bathing condition at 10:00 [*F*(2,18) = 4.124, *p* = 0.034]. The relative power density of the alpha wave of Pz during rest in the mist sauna condition was significantly higher than that of the showering condition at the 11:00 session [*F*(2,18) = 3.838, *p* = 0.041], and during the mist sauna condition, the task was significantly lower than for those in the no bathing condition at the 15:00 session [*F*(2,18) = 4.281, *p* = 0.03] (Figs. 3 and 4). On the other hand, Tsk at the chest in the mist sauna condition was significantly higher than that of the showering condition at 9:00 [*F*(2,18) = 3.575, *p* = 0.049]. In addition, Tsk at the chest in the mist sauna condition was significantly higher than during the no bathing and showering conditions at 15:00 [*F*(2,18) = 4.142, *p* = 0.033] and 16:00 [*F*(2,18) = 3.659, *p* = 0.046] respectively (Fig. 5). The error rate of color-word task of mist sauna condition was lower than those of no bathing condition and showering condition at 14:00 [*F*(2,14) = 8.627, *p* = 0.004] (Fig. 6). However, HRV, BP, AAC, and Tre showed no significant main effects of the bathing method.

## Discussion

In this study, we verified the effects of different methods of morning bathing on human physiological functions and work efficiency during the day. First, we found that HR during rest in the mist sauna condition was lower than that of the no bathing conditions at 11:00. We estimated that the burden of the mist sauna condition was less than no bathing on the cardiovascular system. According to previous studies, the mist sauna produced no significant changes in blood pressure (Miwa et al. 1999; Iwase et al. 2013) as compared to dry sauna bathing (Sudo et al. 1966; Vuori 1998; Watanabe et al. 1994). In particular, Kawahara et al. (2002) compared the effects of a dry sauna bathing with a room temperature of 70 °C and a relative humidity of 10 % and reported that the mist sauna had less effect on the cardiovascular system. While we could not compare our results directly with those, our findings were consistent with the fact that mist sauna has less of an effect on the cardiovascular system.

On the other hand, the relative power density of the alpha wave (α / (α + β)) of Pz during rest in the mist sauna condition was significantly higher than that of the showering condition at 11:00, and during the mental task, the mist sauna condition was significantly lower than the no bathing condition at 15:00. In general, the alpha band (8∼13 Hz) is seen in occipital areas predominantly at the time of awakening, appearing mostly in a state of relaxation, while the beta band (14∼30 Hz) corresponds to a state of high consciousness or excitement. Our results therefore indicated that the mist sauna had a more relaxing effect than showering, evident during the rest period, as well as a greater arousal of consciousness during the task. It indicated that during the rest period, the mist sauna condition was more relaxing than the showering condition in the morning, and the arousal level of the mist sauna condition during the task period was higher than that of the no bathing condition until the afternoon.

In contrast, the error rate of the CWT in the mist sauna condition was lower than for the no bathing condition and showering condition at 14:00. We focused on activation of brain to investigate this result. In this study, we estimated that due to the relative power density of the alpha wave of Pz, the mist sauna condition was significantly lower than the no bathing condition at the task period. Many previous studies have reported that the alpha wave band contributes perceptual facilitation as a result of featured-based visual attention (Kelly and Foxe 2009; Romei et al. 2008). Alpha waves are relatively lower than other waves and are closely related to memory, creativity, and academic performance, because they appear during a stable state of the brain, which seems to be the most receptive in terms of learning and attention (Capotosto et al. 2009; Klimesch and Hanslmayr 2007). We estimated that the activity of the cerebral cortex and level of arousal was higher. Therefore, this might be the source of the low error rate on the task.

Furthermore, the Tsk of the left chest during the mist sauna condition was significantly higher than that during the showering condition at 9:00. The mist sauna condition was also significantly higher than the no bathing and showering conditions at 15:00. We supposed a high skin temperature effect on the relation of the vasodilatation and milder dehydration. Kawahara et al. (2002) reported that the mist sauna resulted in milder dehydration by sweating, indicating its physiologically safe effects; meanwhile, there were higher rates of vasodilatation and sweating as the body temperature rose, demonstrating efficient thermal effects. In addition, Iwase et al. (2013) reported that the mist sauna significantly increased skin blood flow and sweat rate. From these studies, we estimated that the skin temperature in the mist sauna condition was highly maintained in comparison with the other bathing methods, even though a considerable amount of time had passed.

In conclusion, the morning mist sauna suggested that the burden was less in the cardiovascular system and that skin temperature was maintained even though much time had passed. In addition, the work efficiency of the morning mist sauna was better than other bathing condition for the following reasons: It was estimated that low error rate resulted in a high arousal level as the subject conducted the task. This study concludes that a morning mist sauna is safe and maintains both skin temperature compared to other bathing methods. Moreover, it is presumed that the morning mist sauna bathing improves work efficiency comparing other bathing methods during the task period of the day following the morning bathing.
